# Open retropubic prostatectomy for large prostates (Millin Surgery): Why not? It is safe! It is rapid! Complications are few and the learning curve is short!

**DOI:** 10.1590/S1677-5538.IBJU.2016.04.01

**Published:** 2016

**Affiliations:** Professor Associado da Unidade de Pesquisa Urogenital da Universidade do Estado de Rio de Janeiro, RJ, Brasil; Urologista do Hospital da Lagoa Federal, Rio de Janeiro RJ, Brasil

The July-August 2016 issue of the International Braz J Urol presents original contributions with a lot of interesting papers in different fields: Urinary Incontinence, Pyelonephritis, Bladder Cancer, BPH, Prostate Cancer, Renal stones, Renal Cell Carcinoma, Uroginecology, Pediatric Urology and basic research. The papers come from many different countries such as Brazil, USA, Turkey, Italy, Israel, India, China, Iran, Thailand, Egypt, Korea and Colombia, and as usual the editor's comment highlights some papers. We decided to comment 3 papers about a very usual topic in urologic practice: Benign Prostatic Hyperplasia.

Doctor Kobayashi and collegues from Japan performed on page 740 an interesting study about the predictive risk factors of postoperative urinary incontinence following holmium laser enucleation (HoLEP). The authors evaluated 127 patients with benign prostatic hyperplasia who underwent HoLEP. The authors observed that a postoperative urinary incontinence (UI) occurred in 31 patients (24.4%), but it cured in 29 patients (93.5%) after a mean duration of 12 weeks. They concluded that longer enucleation time and increased blood loss were independent predictors of postoperative UI in patients who underwent HoLEP during the initial learning period.

Doctors Wei and Collegues from China performed on page 747 an interesting review study about the Bipolar transurethral enucleation and resection of the prostate (B-TUERP) versus bipolar resection of the prostate (B-TURP) for prostates larger than 60gr. The authors studied 270 BPH patients who underwent B-TUERP and 204 patients who underwent B-TURP for BPH. The authors observed that compared with the B-TURP group, the B-TUERP group had shorter operative time, postoperative bladder irrigation duration and hospital stay, a greater amount of resected prostatic tissue, less postoperative hemoglobin decrease, better postoperative IPSS and Qmax, as well as lower incidences of hyponatremia, urinary sepsis, blood transfusion requirement, urine incontinence and reoperation.

Doctor Pearce and collegues from USA performed on page 757 an interesting study about the Thulium vapoenucleation of large prostates. The authors studied 25 men underwent Thu-VEP, all with prostate volume >75mL. The authors shows that there were 2 intraoperative complications (8%), both cystotomies related to morcellation; Nine patients (36%) experienced a complication, all within 30 days; there were no Clavien III complications. Significant improvements were seen in Qmax, PVR, IPSS, and QoL score at each time interval to 12-months following surgery (all p<0.05). Of 21 patients initially in retention, all were voiding at last follow-up.

The 3 papers that we comment above are very interesting and relevant. I'm a urologist for a developed country and we do not have the same facility to achieve new technologies. I loved the new technologies, but in some cases we need to make some questions. The open retropubic adenomectomy (Millin surgery) is safe, rapid, cheap and had a faster learning curve. In this surgery the transfusion rate 6 %, operation duration is about 88 min., Foley catheterization duration 3.8 days, clinical results at 3 months were: IPSS decrease from 25 to 5 points, quality of life score decrease from 5 to 0.7 points, Qmax increase from 6.5 to 22 mL/sec, PRV decrease from 115 to 7.5 mL (1). In a elegant systematic review, Lucca and collegues shows that in the new techniques for BPH the length of catheter use and estimated blood loss were significantly lower, while the duration of operation was longer than in open prostatectomy (2). In a interesting metaanalysis Li and Collegues shows that the duration of operation was longer for Endoscopic prostatectomy (EP) compared with Open prostatectomy (OP). The resected tissue weight and decrease in hemoglobin were less with EP. EP was associated with fewer blood transfusions. There were no significant differences between EP and OP when comparing other complications (3).

The most important point in the papers about BPH in this number is the postoperative urinary incontinence (UI) that occurred in 24.4% of the cases, with a mean duration of 12 weeks! This is a problem for the patient. This complication do not occur with this frequency during the learnig curve of the Millin surgery. We need to think about the open surgery for large prostates.
